# Re-annotation of the physical map of *Glycine max *for polyploid-like regions by BAC end sequence driven whole genome shotgun read assembly

**DOI:** 10.1186/1471-2164-9-323

**Published:** 2008-07-07

**Authors:** Navinder Saini, Jeffry Shultz, David A Lightfoot

**Affiliations:** 1 Dept. of Plant, Soil and Agricultural Systems: Genomics and Biotechnology Core Facility: Center for Excellence; the Illinois Soybean Center: Southern Illinois University, Carbondale IL, 62901, USA; 2 School of Biological Sciences, Louisiana Tech University, 120 Carson Taylor Hall, Ruston, LA 71272, USA; 3 Biotechnology Centre, Jawaharlal Nehru Krishi Vishwavidyalaya, Jabalpur, India

## Abstract

**Background:**

Many of the world's most important food crops have either polyploid genomes or homeologous regions derived from segmental shuffling following polyploid formation. The soybean (*Glycine max*) genome has been shown to be composed of approximately four thousand short interspersed homeologous regions with 1, 2 or 4 copies per haploid genome by RFLP analysis, microsatellite anchors to BACs and by contigs formed from BAC fingerprints. Despite these similar regions,, the genome has been sequenced by whole genome shotgun sequence (WGS). Here the aim was to use BAC end sequences (BES) derived from three minimum tile paths (MTP) to examine the extent and homogeneity of polyploid-like regions within contigs and the extent of correlation between the polyploid-like regions inferred from fingerprinting and the polyploid-like sequences inferred from WGS matches.

**Results:**

Results show that when sequence divergence was 1–10%, the copy number of homeologous regions could be identified from sequence variation in WGS reads overlapping BES. Homeolog sequence variants (HSVs) were single nucleotide polymorphisms (SNPs; 89%) and single nucleotide indels (SNIs 10%). Larger indels were rare but present (1%). Simulations that had predicted fingerprints of homeologous regions could be separated when divergence exceeded 2% were shown to be false. We show that a 5–10% sequence divergence is necessary to separate homeologs by fingerprinting. BES compared to WGS traces showed polyploid-like regions with less than 1% sequence divergence exist at 2.3% of the locations assayed.

**Conclusion:**

The use of HSVs like SNPs and SNIs to characterize BACs wil improve contig building methods. The implications for bioinformatic and functional annotation of polyploid and paleopolyploid genomes show that a combined approach of BAC fingerprint based physical maps, WGS sequence and HSV-based partitioning of BAC clones from homeologous regions to separate contigs will allow reliable de-convolution and positioning of sequence scaffolds (see BES_scaffolds section of SoyGD). This approach will assist genome annotation for paleopolyploid and true polyploid genomes such as soybean and many important cereal and fruit crops.

## Background

Soybean (*Glycine max*) is the second most valuable crop in the U.S., accounting for $12–17 billion in annual revenue (USDA-NASS Agricultural Statistics 2000–2007). Genomics has had a profound effect on plant biology, but the impact on major crop species such as soybean remains limited to a few marker characterized disease resistant germplasm releases [1,2]. A primary difficulty is that the soybean genome is 4–10 times larger than the model plants *Arabidopsis thaliana*, *Medicago truncatulata *or *Lotus japonicus*. Further, the soybean genome shows evidence of a paleopolyploid origin with gene-rich islands that were highly conserved following duplication [3,4].

Shultz et al., [4] used BAC fingerprint derived contig clone density to estimate that 25–30% of the genome was highly conserved after both duplications, leading to 50–60% of the genome existing in a two- or four-copy state. That conclusion was supported by the gene number in gene families inferred from EST hybridizations to BAC minimum tile paths (MTPs) [5]. Ultimately, Shultz et al., [4] predicted the genome could be resolved into about four thousand segments (each about 150–350 Kbp in size) that differed in copy number per haploid genome. The regions appear interspersed at random, with no evidence for conserved neighbor relationships.

Toward the end of developing a complete map describing where duplicated regions were located, contigs representing each of the genomic segments were rebuilt at high stringency and a minimum number of merges allowed [4]. Despite the high stringency, homeologous regions coalesced to single contigs. Consequently, each contig was measured for the number of BAC clones per unique DNA band. Six clones per unique band in a clone fingerprint was expected, yet regions of 12 and 24 clones per unique band were common. Since homeology could not be distinguished from over-representation of regions in the BAC libraries, contigs were labeled to distinguish their expected copy number. The 2,408 contigs in the 1 to 3,500 series were expected to be largely single copy (1,092 numbers were removed when contig merges were made). The 240 contigs in the 8,000 to 8,999 series were predicted to be present in two copies and derive from the more recent tetraploidy event. Therefore, with further analyses the 8,000 series of contigs were each expected to be separated into two, resulting in 480 different regions [6]. The 406 contigs in the 9,000 to 9,999 series were predicted to be largely coalescences of 4 genomic regions derived from both the genome duplication and hybridization events that produced an octaploid-like genome (though an octaploid-like soybean may never have existed since the two events were separated by millions of years). With further analyses, contigs containing clones from 4 genomic regions were expected to separate into 1,624 different regions. In total, 2,104 multi-copy regions and 2,408 single-copy regions were expected.

DNA markers that anchored the soybean physical map to the genetic map also showed evidence of variation in copy numbers derived from ancient ploidy shifts [4]. All RFLP markers hybridized to clones in two or more contigs. Even the majority (239/363) of microsatellite markers could generate amplicons from clones in two or more contigs. Markers were labeled with an alphabetic suffix, with -a the smallest amplicons, or band, -b the next smallest (sometimes up to -z in cases where many amplicons were found). Alignment of contigs with the genetic map using these anchors was error prone, requiring each marker anchored contig to be shown at each possible location.

To resolve the problem of genetic map placement, microsatellite markers derived from BAC end sequences were used to align contigs with the genetic map [7]. Here, only one outcome was expected, the placement of a single contig to a single location. Maps generated with the markers did show single locations, often in gaps in the existing maps [8]. There were 25,123 BES reads available from the physical map of the 'Forrest' cultivar of soybean that provided about two thousand potential satellite markers. These markers should be enough to locate and orientate every contig at a single map location. These markers, however, cannot separate the polyploid-like regions that are composed of nearly identical homeologous BACs as markers in these regions produce multiple amplicons. New approaches are needed to map these regions correctly.

**Figure 1:**
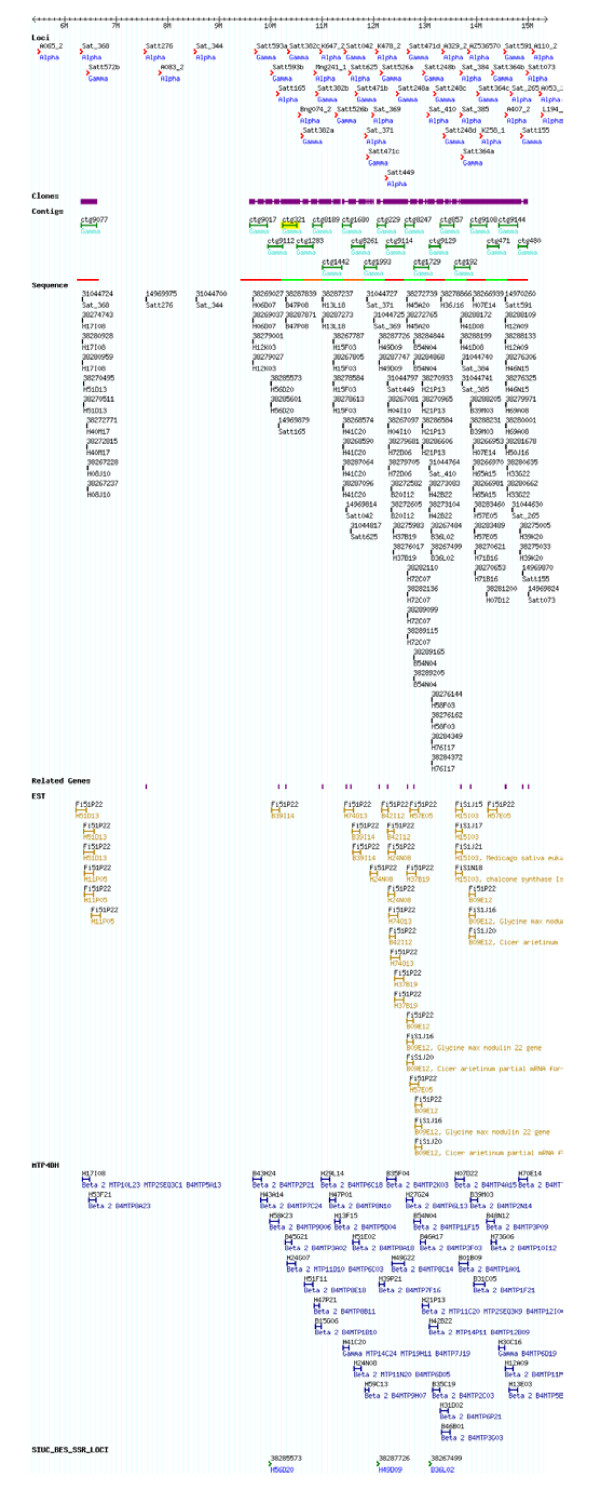
**Description of chromosome LG A1 resources at SoyGD.** The current build 4 representation of 10 Mbp of the 51.5 Mbp LG A1 in SoyGD. Shown are the chromosome (cursor), DNA markers (top row of features); QTL in the region (second row); coalesced clones comprising the anchored contigs (third row); BAC end sequences (fourth row); geneic BES (fifth row); EST hybridizations to MTP2 (sixth row); MTP4 clones (seventh row); BES derived SSR (eighth row); EST hybridizations to build 4 (ninth row); WGS trace file matches from Megablast (tenth and last row). It is recommended readers visit updated site at SoyGD [4] to see a full detailed color version.

SoyGD, based on a distributed annotation systems (DAS) called the generic model organism databases (e.g. GMOD) [9], was developed to show polyploid genomic regions to users (Figure 1). A track for duplications can be inserted into GBrowse [10], however, for highly duplicated genomes, new classifications and ontology for representing large-scale genome duplications had to be developed [4]. Exciting new tools that were developed to compare syntenic regions among genomes [11] demonstrate that the GMOD platform has the potential to display homeology.

The cultivars Forrest and 'Williams 82' provide a large set of useful genomic tools for soybean genomics [6,12,13]. The two cultivars can be thought models in the same way as are cultivars 'Columbia' and 'Landsberg erecta' to *Arabidopsis thaliana*, or 'Mo17' and 'B73' are to *Zea mays*. The soybean community is committed to advancing both resources, with Williams 82 as the lead for a complete genome sequence. In 2007 there were 7.4 million trace sequences at NCBI. Some preliminary sequence contigs with annotations for about 90% of the genome were available. Sequence contigs can be viewed on the genetic map at a new section of SoyGD [4]. These sequence resources represent tools for *in silico *biology that can resolve the physical map and de-convolute the complete genome sequence. Here, these resources were tested for usefulness as tools to determine if the existing contig annotations truly reflect genomic regions that are polyploidy-like, to identify HSVs that can distinguish homeologs within cultivars and to identify HSVs that can distinguish among soybean cultivars.

## Results and discussion

### Genomic regions

**Figure 2:**
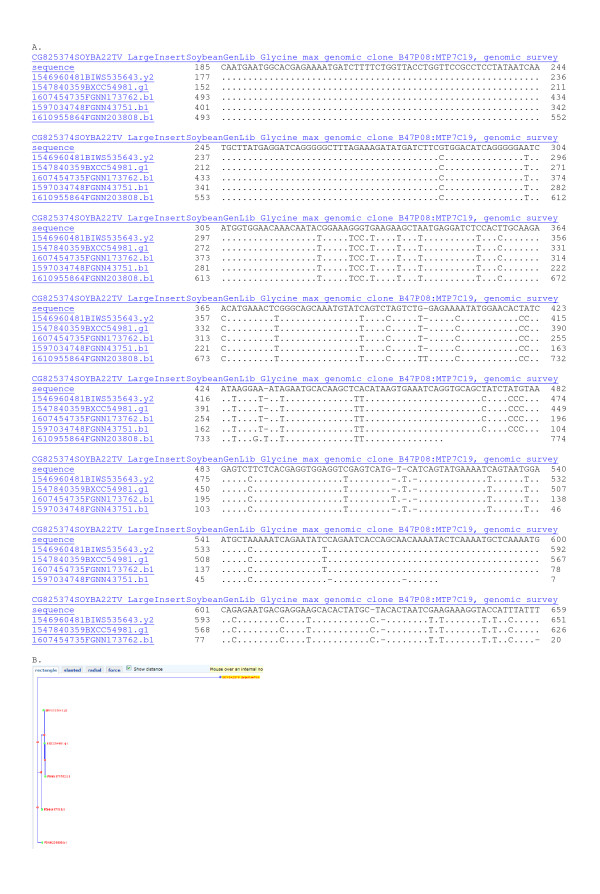
**Analysis of the left hand BES (CG825374) from B47P08 in diploid contig 321.** Sequence analysis supported the inferred diploidized region detected by fingerprints at 90% sequence identity. Panel A: MegaBlast of BES CG825374 against 7.3 million reads with repeat masking gave 5 identical matches. From position 74 to 160 in the BES an extensive set of polymorphisms between Forrest and Williams 82 traces were evident. Panel B: Tree cluster analysis at 90% sequence identity showed the most similar homeologs clustered into 1 set as expected for low copy, diploid region in an inbreeding species.

A MegaBlast of Forrest BES (Table 1) to Williams 82 WGS showed some homeologous regions had diverged significantly and were essentially single-copy (Figure 2). For example, one of the pair of BES from B47P08 (ctg 312; LG A1; Figure 1) showed only 90% identity between Forrest and Williams 82. Only one sequence read matched this BES in Williams 82 trace files, with about 60 SNPs and 6 SNIs. That degree of divergence between cultivars had been reported in sequenced AFLP bands [14,15]. At this degree of divergence though, the Williams 82 sequences may represent a homeologous region. In that case the region being examined was not represented in the Williams 82 WGS. Under-representation may be due to cloning bias against some regions since the WGS is based on a single insertion site in a single high copy number vector.

**Table 1:** Summary of sequence coverage of the three minimum tile paths (MTPs) used for BAC end sequencing made from three BAC libraries.

	MTP4E	MTP4BH	MTP4BH	MTP2 BH	Totals
Vector	pBeloBAC11	pCLD04541	pCLD04541	pCLD04541	na
Insertion site	*Eco*RI	*Bam*HI/*Hind*III	*Bam*HI/*Hind*III	*Bam*HI/*Hind*III	na
Ploidy inferred	diploid	diploid	polyploid-like	mixed	na
Number of clones	3,840	4,608	576	8,064	17,088
Mean insert size (kbp)	175 ± 7	173 ± 7	173 ± 7	140 ± 5	na
Clone coverage^a^	0.7	0.8	0.2	1.4	3.1
BES good reads	3,324	6,772	924	13,473	25,123
BES coverage (Mbp)	2.9	5.0	0.7	9.9	18.5
Predicted gene-like reads	1,512	3,649	498	7,260	12,919

^a ^To calculate the percentage of the soybean genome covered by the clones (clone coverage) in our *Eco*RI- (MTPE) and *Bam*HI or HindIII insert libraries (MTP2BH and MTP4BH), the genome size of soybean was assumed to be 1,130 Mb.

**Figure 3:**
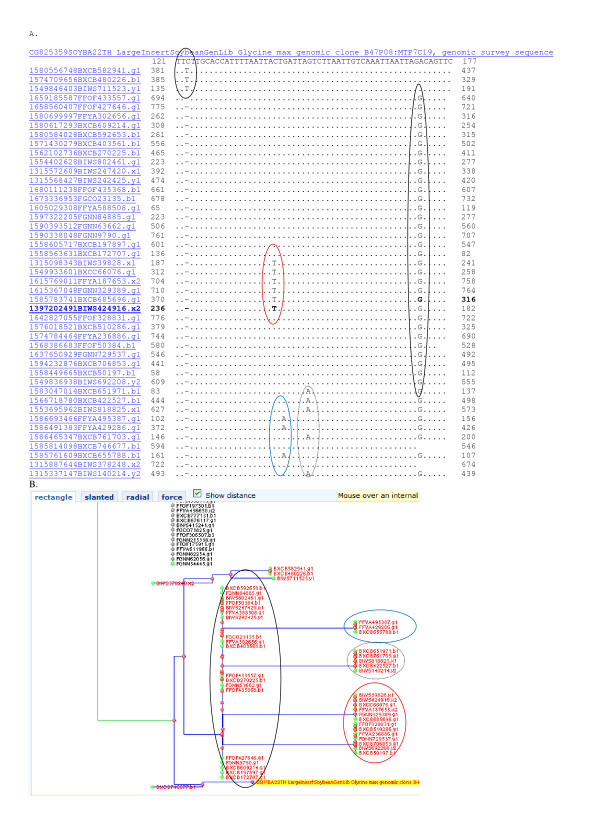
**Analysis of the right hand BES CG825359 from B47P08 in diploid contig 321.** Sequence analysis suggested the inferred diploid contig contained a short octaploid-like region not detected by fingerprints. Panel A: Analysis of the BAC end from B47P08 detected an octaploid-like region on the end of ctg321. MegaBlast of CG825359 against 7.3 million reads with repeat masking gave 3 nearly identical sequences and 42 homeologus sequences. Among the 3 nearly identical matches a SNP was evident at nucleotide position 123 (circled). From positions 141 to 177 a set of four SNH polymorphisms were evident (circled in red at 141, blue at 143, dotted 148 and black at 170) that separated the homeologs into 4 groups. Panel B: Tree cluster analysis at 90% sequence identity showed the most similar homeologs clustered into 4 sets as expected for octaploid-like region in an inbreeding species. Clusters sharing SNHs are circled in red at nucleotide 141 (3 reads), blue at 143 (five reads), black dotted 148 (13 reads) and black solid at 170 (21 reads).

Examination of the second BES from B47P08 (ctg 312; LGA1; Figure 1) showed three sequences with 99% identity between Forrest and Williams 82 (Figure 3). The differences represent probable SNPs between cultivars. However, in addition there were 43 sequences with >95% identity. Those homeologous sequences could be clustered into 4 different groups based on SNPs among HSVs (H-SNPs). Contig 321 appears to be octaploid-like at one end and diploid-like at the other end. Contigs of this type are dangerous to merge with neighboring contigs, as eight different regions might match, only one of which would be correct.

**Figure 4:**
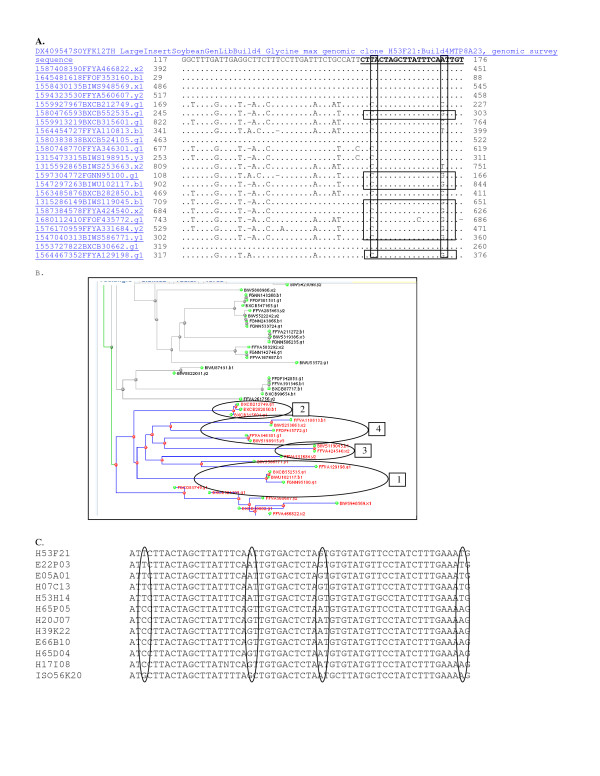
**Analysis of sequence identity between WGS trace files and the BES DX409547 from H53F21 in octaploid-like contig 9077.** Sequence analysis showed the region was octaploid-like as inferred by fingerprints. Panel A: MegaBlast of "H53F21_Build4MTP8A23_gi89261445_4" against 7.3 million reads with repeat masking gave 7 identical matches among 24 homeologous sequences. Cluster 1 was composed of traces ending in 822,160,569,607,662,749 and 105 that shared A at position 172 (boxed). HSVs were evident among the 4 clusters inferred. Cluster 2 was composed of traces ending in 749, 850,601 and shared C at position 172. Cluster 3 was composed of traces ending in 100, 117 and 535 that shared G at position 172. Cluster 4 also had G at that position and was heterogeneous being composed of clones with different HSVs (traces ended in 813, 663, 772, 301 and 891). Panel B: Treecluster analysis showed the most similar homeologs clustered into 4 or more separate sets as expected for an octaploid-like region (circled). Panel C: The sequences found among eight BACs that overlapped H53F21 in contig 9077were re-sequenced. A set of SNHs separated the BACs into two of the four groups expected to be present in contig 9077 from the data shown in Panel A. The region shown corresponds to the 152–210 bp of the BES encompassing the 22 bp region in bold and underlined in Panel A that contained two HSV.

**Figure 5:**
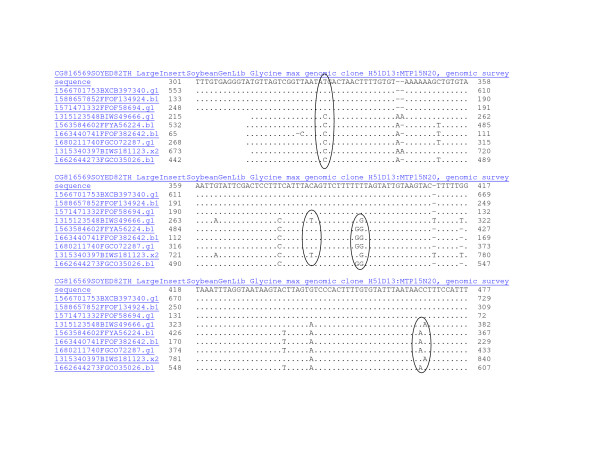
**MegaBlast analysis showing sequence identity to WGS reads using the BES CG816569 from H51D13 in octaploid-like contig 9077.** Sequence analysis only partly supported the inference that the contig was a product of four coalesced regions. Shown is an alignment of 301–477 bp of the BES "H51D13_Build4MTP8A23_gi89261445_4" against 7.3 million reads with repeat masking that gave 3 identical matches among 9–10 homeologous sequences. Cluster 1 was composed of traces ending in 340, 924 and 694 that shared T rather than C at position 330 (circled). Cluster 2 was composed of clones ending in 666 and 123 and shared T rather than C at position 375. Cluster 3 was composed of clones ending in 694, 224, 642, 287 and 026 that shared GG at position 385. The homeologs clustered into 3 separate sets, one less than the number expected for an octaploid-like region in an inbreeding species.

Contig 9077, also from LG A1 (Figure 1), is a potentially octaploid-like contig. Examination of the four BES from H53F21 (Figure 4 and not shown) and H51D13 (Figure 5 and not shown) showed evidence to support the octaploid-like nature of the contig. For example, at H53F21 there were three sequences with 99% identity between Forrest and Williams 82 (Figure 4). The differences among these three and the Forrest BES may represent SNIs between cultivars (few or no SNPs). However, in addition there were 22 sequences with >90% identity. Those clearly homeologous sequences could be clustered into 3 different groups based on H-SNPs.

At BES H51D13 (Figure 5), about 300 Kbp away on LG A1 (Figure 1), the genomic region was less well represented among trace files. There were three sequences with 99% identity between Forrest and Williams 82 (Figure 5). The differences represent probable SNIs between cultivars (few or no SNPs were found). However, in addition there were 5–6 different sequences with >90% identity. Those clearly homeologous sequences could be clustered into 3 different groups based on H-SNPs.

Fourteen BAC clones were chosen from contig 9077 and used for PCR amplification of the BES. Sequencing these amplicons revealed two sequences, each representing one of two homeologs mixed together throughout the contig (Figure 4; Panel C). The A type and the G type were present but the T type and the C type found in WGS were not present. Therefore, the third and fourth homeologs predicted to be in the contig by WGS to BES alignments could not be distinguished by the >600 bp of DNA sequence. The G type and A type clones can each be used to form a new contig. The SNHs will be used to split ctg9077 (Figure 1) in two. Map locations for the split contigs may be determined if cultivar differences can be found linked closely to the HSVs among common mapping population parents.

Of further note, micro-satellite marker Sat_368 anchoring contig 9077 was on the G-type clone ISO56K20. Sat_368 did not appear to have any close homeologs (Additional File 1). Therefore, the octaploid-like regions can be quite heterogeneous across contig-sized regions and suggests diploidization acts on regions less than the size of a BAC clone.

**Figure 6:**
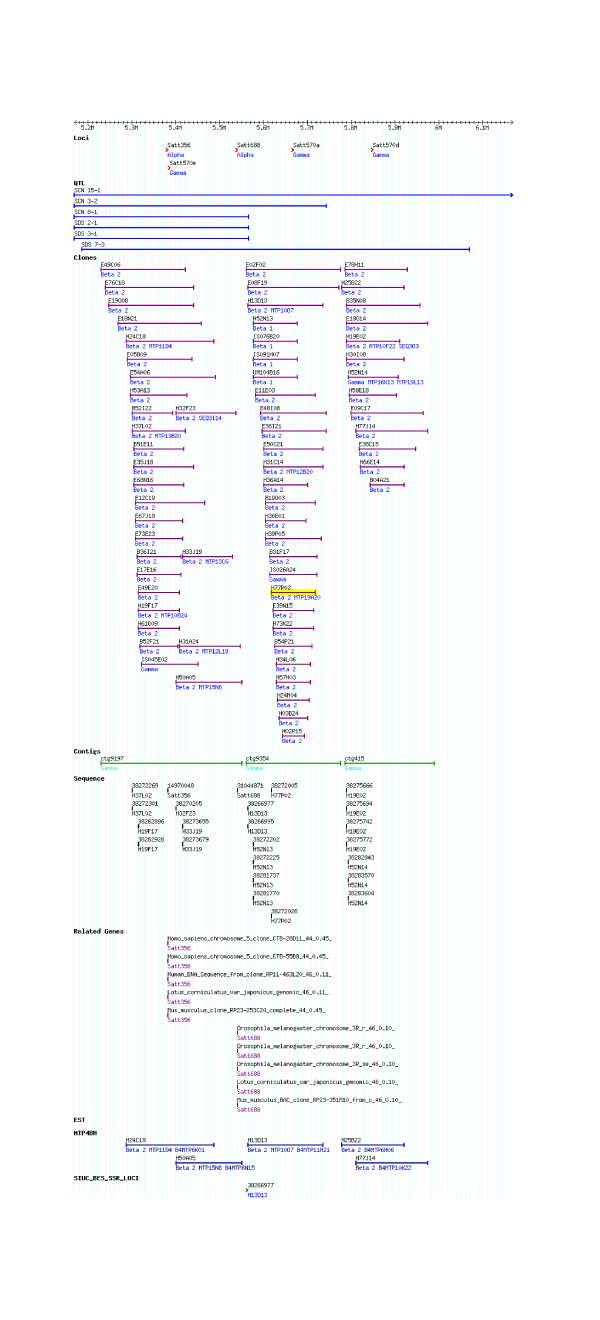
**Description of a region of chromosome LG G at SoyGD encompassing the examples shown.** The current GMOD representation the build 4 representation of 1.0 Mbp of the 51.5 Mbp LG G in SoyGD. Shown are the chromosome (cursor), DNA markers (top row of features); QTL in the region (second row); coalesced clones comprising the anchored contigs (third row); BAC end sequences (fourth row); geneic BES (fifth row); EST hybridizations to MTP2 (sixth row); MTP4 clones (seventh row); BES derived SSR (eighth row); EST hybridizations to build 4 (ninth row); WGS trace file matches from Megablast (tenth and last row). It is recommended readers visit updated site at SoyGD [4] to see a full detailed color version and a build 5 view. The gaps between contigs will be filled in build 5 by contig merges suggested by BES-SSRs and contig end overlap data.

**Figure 7:**
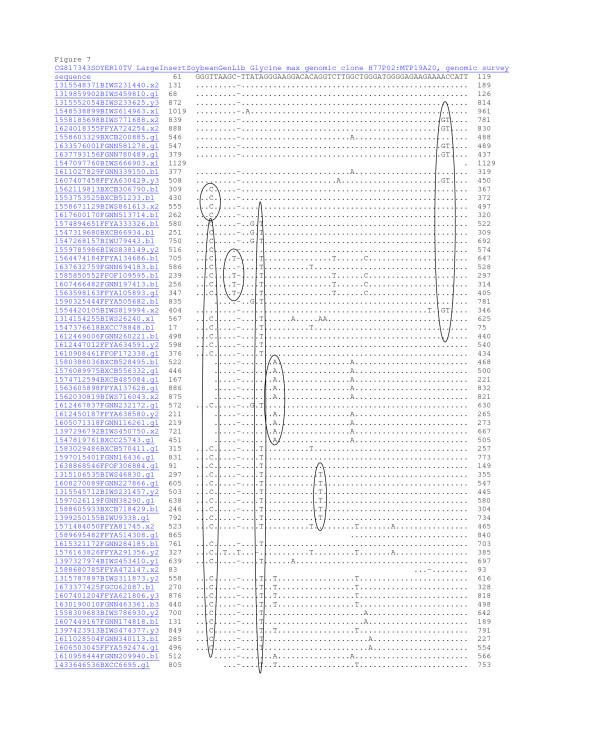
**MegaBlast analysis showing sequence identity to WGS reads using the BES CG817343 from H77P02 in octaploid-like contig 9354.** Sequence analysis strongly supported the inferred octaploid-like structure for the region coalesced by fingerprints. MegaBlast against 7.3 million reads with repeat masking gave 3 identical matches (trace files ending 440, 810 and 625) among 68 homeologous sequences. Homeolog cluster 1 was composed of traces ending in 688, 254 278, 421 and 994 that shared GT instead of AC at position 114–115 (circled). Cluster 2 was composed of clones ending in 790, 233, 613 and 714 shared C instead of T at position 64 but not the G of Cluster 3. Cluster 3 was composed of clones ending in 326, 934, 443, 682 and 172 that shared G instead of A at position 73. Clusters 4, 5 and 6 contained more than 4 reads suggesting this region may be over-represented in the WGS collection or contain repetitive DNA.

A similar pattern was observed on LG G (Figure 6). Examination of the pair of BES from H77P02 (Figure 7) in Contig 9354 shows that four genomic regions potentially coalesced and indicates the octaploid-like nature of the contig. For example, at BES SOYFK12TH there were three sequences with 99% identity between Forrest and Williams 82 (Figure 7; Panel A). The differences represent probable SNIs between cultivars (no SNPs). In addition, there were 65 homeologous sequences with >95% identity which could be clustered into 4–9 different groups based on H-SNPs (Figure 7; Panel B).

**Figure 8:**
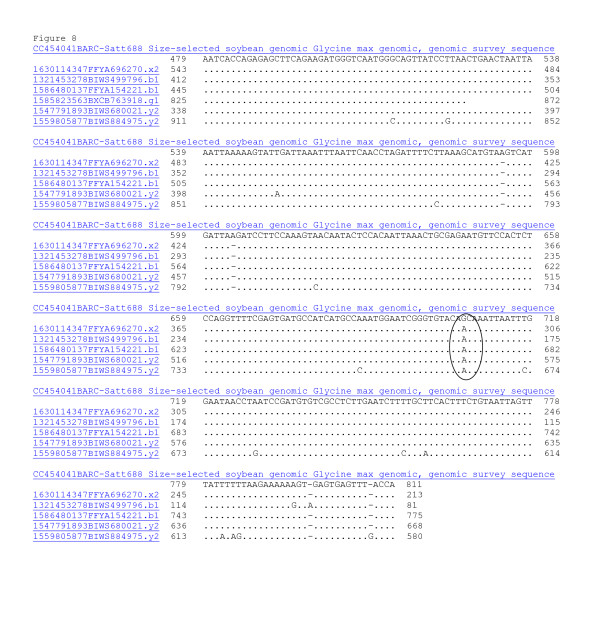
**MegaBlast analysis showing sequence identity to WGS reads using the sequence of the satellite marker BARC-Satt688 (CC454041) that anchored the BES from H77P02 (CG817343 and CG817333) to contig 9354.** MegaBlast against 7.3 million reads with repeat masking gave 4 nearly identical. If the few differences were HSVs then there might be 3 homeologous sequences. Each homeolog was represented by a single read suggesting this region is under-represented in the sequence database. The ATT repeat that formed the microsatellite polymorphism was at 360–418 bp (not shown) and was 2 bp shorter in Williams 82 than Forrest. A potential SNP between Forrest and Williams 82 is circled (SIUC-SNP_Satt688).

Even the satellite marker Satt688, anchoring BAC H77P02 to contig 9354 and the genetic map showed evidence for conserved homeologs (Figure 8). There were four different HSV sequences with 99% identity. Differences among homeologs were evident as 1 (trace file -021), 2 (trace file -796) and 11 (trace file -795) probable SNPs compared to the trace file -270. None of the four genomic regions was adequately represented among the trace files, suggesting a cloning bias against each. An alternate hypothesis is that the sequence variations are errors in the reads of a single region, which is unlikely given the genomic context of the marker.

### Whole genome comparisions

Because BAC clones were selected from the MTP to generate BES, there are many fewer BES from polyploid-like regions than diploidized regions (Table 2). Nonetheless, there are sufficient numbers to examine the usefulness ploidy annotations found at SoyGD. Allowing for 90% identity, the mean number of homeologs inferred from the BES to WGS matches was 8.4 for octaploid-like contigs; 4.5 for tetraploid-like contigs and 1.5 for diploidized contigs. Those means were in good agreement with those inferred from clones per unique band metric used to label contigs in the physical map as potentially repeated and octaploid-like [4]. At 95% identity there were about half as many matches on average, suggesting divergence following the most recent genome duplication has been less than after the first event. At 98 and 99% identity there was evidence for sequence pairs that share significant identity in the polyploid-like regions but not in the diploidized regions.

**Table 2:** Characteristics of ploidy among the three groups of contigs with BESs.

	Diploid-like	Tetraploid-like	Octaploid-like
Number of sequences^a^	20,548	1,070	3,506
Mean number of homeologs^b ^detected at 90% identity	1.5	4.5	8.4
Mean number of homeologs detected at 95% identity	1.3	2.4	4.1
Mean number of homeologs detected at 98% identity	1.2	2.1	2.6
Mean number of homeologs detected at 99% identity	1.1	1.9	2.3

Megablast searches and SNP analysis were used to identify the mean number of homeologous groups of sequences above three identity percentages. Most BES from ctgs 1–3500 showed matches to regions that were single copy, however a significant number of regions from contigs formed from 2 or 4 coalesced regions could be detected at 90, 98 and even 99% sequence identity.

^a ^Two hundred sequences were selected from these groups for visual scoring of the BLAST searches.

^b ^The BAC libraries were each constructed from DNA derived from twenty five different seedlings of cultivar Forrest, bulked after single pod selection at F2:5 from F2:6 to F2:13, but otherwise inbred. Therefore, less than 7% heterogeneity should have remained from 'Dyer' and 'Bragg' the parents of Forrest.

### Genes and markers

BAC end sequences anchored to a robust physical map are important tools for genome analysis. BES have been developed from MTP2BH, MTP4BH and MTPE4 (Table 1). Enquiries to GenBank nr and pat databases identified 12,919 potentially geneic homologs. Analysis of the locations of the inferred genes showed evidence of gene rich islands on each chromosome (Figure 1; Figure 6).

Eighty one homologs of DNA markers found in genetic maps were detected in the BES, i.e. forty two BES's contained sequence highly homologous (over 80–341 bp from e^-30 ^to e^-300^) to 80 different genetic markers (20 RFLPs, 61 microsatellites), or about 4% of the markers with sequences in GenBank. About three thousand new microsatellite markers were identified within the whole BES collection.

SNPs among the HSVs were found in nearly every BES examined. SNIs among the HSVs were found among 24% of sequences (Figure 2, 3, 4, 5, 6, 7, 8). Clones in plates 11 and 12 were re-sequenced and so have 2 records for each BAC end in GenBank. Re-sequenced clones help determine the sequence error rate and greatly facilitate SNP detection. Along with the few clones tested directly by mapping (data not shown), about 67.5% of SNPs and SNIs detected in single pass sequence are expected to be validated [8].

## Conclusion

The comparison of Forrest and Williams 82 sequences represents a powerful tool for soybean geneticists. There are abundant SNPs and SNIs among the sequences, with many linked to predicted gene sequences (Table 1). The high frequency of single nucleotide changes between genomic regions of soybean cultivars has been reported previously [14,15] and stands in contrast to the very low frequency between ESTs [16]. Clearly, further investment in genomic SNP identification is called for. MTP BES [17] make an excellent starting point, providing markers spaced at regular intervals in the genome.

In comparison to SNIs, indels larger than 2 bp are very rare. This bias against indels may explain why RFLPs and AFLPs are rare in soybean [3,14]. Further the scarcity of indels will have contributed to the inability of FPC to separate BACs into different contigs, once their sequence identity exceeded 90% [4,18]. The use of SNPs and SNIs to characterize BACs will improve contig building methodology. For example, plate 13 of MTP4BH was developed from just 6 octaploid-like contigs by picking redundant clones from putatively octaploid-like contigs [4]. This set of 748 sequences should resolve into 48 regions when the genome sequence is properly de-convoluted.

Bioinformatic and functional annotation of polyploid genomes can be greatly improved using a combination of BAC fingerprint based physical maps, WGS sequence and HSV partitioning of BAC clones in polyploid regions. The separation of contigs will allow the de-convolution of sequence and allow whole genome annotation in polyploids. Preliminary results from stringent BLAT analysis of BES to sequence scaffolds can be viewed at SoyGD [4]

Major challenges will have to be overcome in assigning function to the duplicated regions. Reverse genetic approaches like gene silencing and mutation would be expected to be effective only in certain small gene families and particular genomic regions. Gene silencing should work when duplicated genes of redundant function are close enough in sequence to be inhibited by the same probe [19]. During over-expression [20], the co-suppression response of the endogenous gene family will have to be considered. Will co-suppression actually reduce the activity of the members of the gene family in patterns not predicted by the experimenters? In the case of the identification of mutations for loss of function by TILLING [21-23], the functions of the homeologous genes must have been sufficiently diverged over evolutionary time for success to be expected. Secondly, no aneupleurotic pathways with functional redundancy must exist. The physical map should be used to guide these approaches. A complete map of homeologous regions can help identify genes in regions likely to be unique, single copy, and others likely to be redundant in 2 or 4 copies like an allo-polyploid.

## Methods

### Source of sequences

Forrest genome resources used included all three MTPs described in Table 1. There were 13,473 BES reads from MTP2 (CG826126 to CG812653). There were 7,700 BES reads from MTP4BH (DX406713 to DX414412) and 3,324 reads from MTP4E (ER962965 to ER966289). After trimming, the mean read length for these BES was about 736 bp. The total sequence generated was 18.5 Mbp, or about 2% of the soybean genome. There were 9,386 paired, forward and reverse reads.

At the time of enquiry (mid 2007), there were 7.4 million reads of Williams 82 genome reported in the trace sequence section of NCBI [29]. The total amount of sequence was 6,000 Mbp, about six fold the soybean genome. Most were paired forward and reverse reads from 2–3 kb inserts. These sequences were not trimmed and most contained 50–60 bp of sequence from pUC18 at the start of the sequence. About 36,000 reads had another tract from pUC18 at the end of the sequence.

### *In silico *polymorphism detections

MegaBlast enquiries were made of the *Glycine max *WGS database using individual BES [29]. Criteria set were; database *Glycine max*-WGS; hits computed 250; all low complexity filter selected; expect was set to 10; word size was set to 32 or 64; percent identity used was normally 90% though 99%, 98%, 95%, and 85% were manually tested in instances noted in the results.

Results were assembled into groups of 100 by expected copy number and 600 were examined by a manual editor. Distance trees of the results were selected (some some typical result trees were captured as screen shots (Figures 2, 3, 4). "Show multiple alignment" was selected from the root of the tree. Multiple alignments were examined for the presence of HSVs, SNPs, SNIs and SSRs and illustrative examples used to make Figures 2, 3, 4, 5, 7 and 8. For the means in Table 2, multiple alignment was selected directly and the results automatically recorded.

### *In vitro *HSV polymorphism detection

BACs from homeologous regions that assembled into single contigs were picked from BAC library master plates. DNA was extracted as previously described [4,17]. Primers were designed from within the BES to encompass HSV. Settings used for primer design were T_m _55° ± 1°C, amplicons 100–500 bp, primer length 20 ± 2 bp. No constraints on GC% were set to avoid potential bias against the AT rich regions of the soybean genome. Repeated DNA amplicons (mini-satellites, transposons etc.) were filtered out by Blast searching, unlike Shultz et al., [7]. Primers were obtained from Sigma Genosys (Woodlands, TX).

Polymerase chain reaction (PCR) was performed in a PE 9700 (Boston, MA). An initial 95°C denaturation for 5 min was followed by 30 cycles of 95° for 30 s, 55° for 30 s, and 72° for 30 s. After PCR was complete, gel electrophoresis was performed in a 2% (w/v) agarose gel or a 4% (w/v) PAGE stained with ethidium bromide and amplicon documented using a BioRad GelDoc (Hercules, CA) system. Bands were isolated in pGEM3T. SNP polymorphism was identified by DNA sequencing of PCR amplicons following plasmid isolation using a CEQ2000 (Beckman Coulter, Fullerton, CA).

### Annotation and map representation

All potential SNPs, SNIs and microsatellites that distinguish either cultivars or homeologs were named with the SIUC_ suffix (at each database entry and first mention in the text) followed by N-, I- or S- prefix. For cultivar polymorphisms this was followed by the motif and BAC of origin. For HSVs the letter H- was suffixed, then followed by the motif and BAC of origin. In contrast, earlier markers were assigned a sequential number [30-33]. The altered naming convention used here was designed to help users find the clone of origin in the physical map. All potential markers will be shown at SoyGD in the BES_SSR, BES_SNP or BES_SNI track (not shown). Markers that have been located in the genetic map by DNA polymorphism scored in RIL populations will be shown on the locus track.

## Abbreviations

BAC: bacterial artificial chromosome; BES: BAC end sequence; bp: base pair; contig: contiguous set of overlapping clones; EST: expressed sequence tag; HSV: homeolog sequence variant; LIS: legume information system; MTP: minimum tiling path; NCBI: National Center for Biotechnology Information; RFLP: restriction fragment length polymorphism; SNH: single nucleotide polymorphism between homeologs; SNI: single nucleotide insertion; SNP: single nucleotide polymorphism between alleles; SSR: simple sequence repeat.

## Authors' contributions

DAL and NS conceived of the study and executed all of the analysis. JS classified the contigs for ploidy based on clones per unique band. DAL drafted the manuscript. NS and JS provided critical review, interpretation of results and funding. All authors read and approved the final manuscript.

## Supplementary Material

Additional File 1 The WGS trace files homeologous to marker sequence Sat_368. No evidence for multiple homeologs was found. Click here for file
