# Supplementary material for: Re-annotation of the physical map of Glycine max for polyploid-like regions by BAC end sequence driven whole genome shotgun read assembly
Source: BMC Genomics. 2008 Jul 7;9:323. doi: 10.1186/1471-2164-9-323 (PMC2478686; doi:10.1186/1471-2164-9-323)
Supplement: Additional File 1 — The WGS trace files homeologous to marker sequence Sat_368. No evidence for multiple homeologs was found. [file 1471-2164-9-323-S1.doc]

Additional File

CC453894BARC-Sat_368 1 TTACAAACTTACTCTTTCTAAAAAAAATAGGTGAATA-TAAATTAGTAAACTTACACACA 59

1625860308FFYA672472.x2 346 .....................................-...................... 404

1876391758FGNN847759.b1 68 .....................................-...................... 126

1673343863FGCO32829.b1 706 .....................................T...................... 647

1315458078BIWS185715.x1 795 ..................-...................... 756

1922253873FOSU232534.b1 629 ...............C........C............-...................... 571

1558488056BXCB133516.b1 9 ........-...-...................... 41

1547190473BIWU44991.g1 421 .....................................-...................... 363

1547052801BIWS600364.g1 505 .....................................-...................... 563

1571500768FFYA268351.g1 371 .....................................-...................... 313

1397621599BIWS481084.y1 347 .....................................-...................... 289

1397203620BIWS424137.x2 601 .....................................-...................... 659

1563471222BXCB265892.g1 312 .....................................-...................... 254

CC453894BARC-Sat_368 60 TTTATTAATATACACTCACTTA-TTTTTCAAGAGTAAATTAGAAAGGTACACTTTAAGTG 118

1625860308FFYA672472.x2 405 ......................-..................................... 463

1876391758FGNN847759.b1 127 ......................-..................................... 185

1673343863FGCO32829.b1 646 ......................T..................................... 587

1315458078BIWS185715.x1 755 ......................-..................................... 697

1922253873FOSU232534.b1 570 ......................-..................................... 512

1558488056BXCB133516.b1 42 ......................-..................................... 100

1547190473BIWU44991.g1 362 ......................-..................................... 304

1547052801BIWS600364.g1 564 ......................-..................................... 622

1571500768FFYA268351.g1 312 ......................-..................................... 254

1397621599BIWS481084.y1 288 ......................-..................................... 230

1397203620BIWS424137.x2 660 ......................-..................................... 718

1563471222BXCB265892.g1 253 ......................-..................................... 195

CC453894BARC-Sat_368 119 TA-TTTT-AAGATAAACATATACTCCTGCTAAAAAAAATATGTATAAATTAAAAAATCCA 176

1625860308FFYA672472.x2 464 ..-....-.................................................... 521

1876391758FGNN847759.b1 186 ..-....-.................................................... 243

1673343863FGCO32829.b1 586 ..-....A.................................................... 528

1315458078BIWS185715.x1 696 ..-....-.................................................... 639

1922253873FOSU232534.b1 strain ..T....-.....................................GGGGGG......... 453

1558488056BXCB133516.b1 101 ..-....-.................................................... 158

1547190473BIWU44991.g1 303 ..-....-.................................................... 246

1547052801BIWS600364.g1 623 ..-....-.................................................... 680

1571500768FFYA268351.g1 253 ..-....-.................................................... 196

1397621599BIWS481084.y1 229 ..-....-.................................................... 172

1397203620BIWS424137.x2 719 ..-....-.................................................... 776

1563471222BXCB265892.g1 194 ..-....-.................................................... 137

CC453894BARC-Sat_368 177 TATATATATATATATATATATATATATAATATAAC-AAAAATATAAATATACATTACTTT 235

1625860308FFYA672472.x2 522 ...................................-........................ 580

1876391758FGNN847759.b1 244 ...................................-........................ 302

1673343863FGCO32829.b1 527 ...................................-........................ 469

1315458078BIWS185715.x1 638 ...................................-........................ 580

1922253873FOSU232534.b1 452 ...................................-........................ 394

1558488056BXCB133516.b1 159 ...................................-........................ 217

1547190473BIWU44991.g1 245 ...................................-........................ 187

1547052801BIWS600364.g1 681 ...................................-........................ 739

1571500768FFYA268351.g1 195 ...................................-........................ 137

1397621599BIWS481084.y1 171 ...................................-........................ 113

1397203620BIWS424137.x2 777 ...........................T.......C........................ 836

1563471222BXCB265892.g1 136 ...................................-........................ 78
